# Supplementary material for: A semi-automated machine-learning based workflow for ellipsoid zone analysis in eyes with macular edema: SCORE2 pilot study
Source: PLoS One. 2020 Apr 30;15(4):e0232494. doi: 10.1371/journal.pone.0232494 (PMC7192485; doi:10.1371/journal.pone.0232494)
Supplement: Attachment — Submitted filename: Response to Reviewers.docx [file pone.0232494.s001.docx]

January 30, 2020

To: Jeorg Heber, PhD

PLOS ONE

RE: A Semi-Automated Machine-Learning Based Workflow for Ellipsoid Zone Analysis in Eyes with Macular Edema: SCORE 2 Pilot Study

Ref.: PONE-D-19-34185

Dear Jeorg Heber,

We appreciate the thoughtful reviews to enhance the quality of our manuscript for consideration of acceptance in PLOS One. Please see our responses below which address each of the points outlined by the reviewers.

**Review Comments to the Author

Reviewer #1:**

1. A very well structured manuscript, technically sound, with data that support the conclusions
2. The statistical analysis described is thorough.
3. The lack of EZ assessment at baseline scans defeats the purpose of using EZ integrity as a prognosticator of final visual outcome:
   As described in the introduction, one of the reasons why EZ integrity is often assessed is its potential use as a predictive factor of the final visual outcome. In the present study, by converting cross sectional images into en face, hemorrhage or edema-associated signal blockage prevented the assessment of EZ integrity at baseline. (>90% of baseline images were ‘ungradable’)
   Hence, as described, EZ integrity of only SD-OCT images at months 1, 6, and 12 was assessed, analysed and quantified in this study. Consequently, if a semi-automated machine learning system is used for the assessment of EZ integrity in en face images, the only prognostic information one can derive comes from the EZ integrity after intravitreal anti-VEGF treatment (at 1st month)

**Reply:** No changes were made. Please see the discussion section (page 13, line 282-298). The inability to accurately assess the EZ on en face thickness maps due to reduced outer retinal signal intensity from blockage by hemorrhage and fluid is an inherent challenge in retinal vein occlusion. Without histopathological correlates it is difficult to know whether EZ defects on SD-OCT are artifacts resulting from signal blockage or loss of photoreceptors secondary to disease. However, our approach of assessing month 1 EZ integrity with subsequent visual acuity matches other studies referenced in the manuscript:

Chan EW, Eldeeb M, Sun V, et al. Disorganization of Retinal Inner Layers and Ellipsoid Zone Disruption Predict Visual Outcomes in Central Retinal Vein Occlusion. Ophthalmol Retina 2019;3:83-92.

Tang F, Qin X, Lu J, Song P, Li M, Ma X. Optical Coherence Tomography Predictors of Short-Term Visual Acuity in Eyes with Macular Edema Secondary to Retinal Vein Occlusion Treated with Intravitreal Conbercept. Retina 2019.

Fujihara-Mino A, Mitamura Y, Inomoto N, Sano H, Akaiwa K, Semba K. Optical coherence tomography parameters predictive of visual outcome after anti-VEGF therapy for retinal vein occlusion. Clin Ophthalmol 2016;10:1305-13.

**Reviewer #2:**

The paper by Tyler Etheridge et al. entitled 'A Semi-Automated Machine-Learning Based Workflow for Ellipsoid Zone Analysis in Eyes with Macular Edema: SCORE 2 Pilot Study' is an interesting study on the evaluation of the combination of a customized workflow with KNIME and machine learning tool for Fiji plugin: Trainable Weka Segmentation. The authors developed the customized workflow with semi-automated optical coherence tomography measurements and examined its agreement and reliability. And then, they found their new approach was reliable and time-saving. The study is well constructed. The obtained results will be of interest to researchers in the field. Their handy method using KNIME and Fiji plugin could be applied to other retinal diseases at a low cost. And I think the study has great potential not only in academic research but also in daily clinical practice.

I have few concerns,
(1) Page 7, line 170-172; what kind of dataset did the authors use for the learning process? From entire SCORE2 data? Or other normal subject data? And how much data did the authors use for training? Did the authors check if the performance reached the plateau or not?

**Reply:** No changes were made. Please see the methods section (page 8, line 175-179). The authors selected en face thickness maps that represented the inherent variability of the total dataset analyzed (184 scans). We chose approximately 10% of the dataset (12 en face thickness maps) for classifier training. Areas of normal EZ and EZ defects were manually traced using the Trainable Weka Segmentation plugin of Fiji as described by Arganda-Carreras et al.*. The authors sought to maximize the classifiers identification of regions of interest and minimize non-region of interest (false-positive) identification. A reliable and accurate classifier was trained with the initial 12 thickness maps, more would have been used if necessary, as is customary in such training protocols.

* Arganda-Carreras I, Kaynig V, Rueden C, Eliceiri KW, Schindelin J, Cardona A, et al. Trainable Weka Segmentation: a machine learning tool for microscopy pixel classification. Bioinformatics. 2017;33(15):2424-6. doi: 10.1093/bioinformatics/btx180. PubMed PMID: 28369169

(2) Page 11, line 247-255; how long did it take for the training process?

**Reply:** No changes were made. The authors did not time the classifier training. However, after generation of the en face thickness maps and selection of images representing the heterogeneity of the image dataset, the training of the classifier required minimal time compared to manual annotations (20-30 minutes to obtain the first, most-reliable classifier with appropriate settings).

(3) Page 9, line 210-218; how did the authors judge whether each image could go through the analysis or not. Manually by graders or semi-automated with a program? Did 'gradable' mean manually measured? Did only images that could be manually measured go through semi-automated measurements? If the judgment is done by a human, not by a semi-automated program, then I suggest discussing this essential process in terms of the semi-automated approach limitation.

**Reply:** The following changes were made (page 14, line 308-312). **“**The selection of scans for analysis of the EZ was performed by expert graders and was based on the SCORE2 SD-OCT grading protocol. However, all scans deemed of sufficient quality for EZ assessment (i.e. the RPE was visible) were evaluated by both the semi-automated and manual approaches. Future studies may examine the application of machine-learning for image selection.”

(4) Page 10, line 231-233 (Figure 3A); the authors evaluated the reliability with Bland-Altman plot in addition to intraclass correlation coefficient. But, I guess intraclass correlation coefficient is enough to evaluate both the reliability and agreement (ref. Terry K. Koo, Mae Y. Li. A Guideline of Selecting and Reporting Intraclass Correlation Coefficients for Reliability Research. J Chiropr Med. 2016 Jun; 15(2): 155–163. Published online 2016 Mar 31. doi: 10.1016/j.jcm.2016.02.012 PMCID: PMC4913118. Correction in: J Chiropr Med. 2017 Dec; 16(4): 346. Pubmed Central PMCID: PMC5731844).

**Reply:** No changes were made. The authors sought to perform a rigorous comparison of the two approaches and subtle differences exist between reliability and agreement, which is described by Kottner et al.*.

*Kottner J, Streiner DL. The difference between reliability and agreement. J Clin Epidemiol. 2011 Jun;64(6):701-2; author reply 702. doi: 10.1016/j.jclinepi.2010.12.001. Epub 2011 Mar 16. PMID: 21411278.

(5) For the researchers and clinicians who want to use the workflow and semi-automated methods in their settings, is it possible to show the link of the programs or training dataset? If it's possible, it must be helpful for our fields.

**Reply:** The following changes were made to the Data Availability Statement. “The KNIME workflow is available at https://hub.knime.com/tetheridge/spaces/Public/latest/Raw_Thickness_Maps. The KNIME workflow protocol and training dataset is available at https://doi.org/10.6084/m9.figshare.11774577.”

Sincerely,

Amitha Domalpally, MD, PhD

Fundus Photograph Reading Center

Department of Ophthalmology and Visual Sciences

University of Wisconsin School of Medicine and Public Health
