# Supplementary material for: A semi-automated machine-learning based workflow for ellipsoid zone analysis in eyes with macular edema: SCORE2 pilot study
Source: PLoS One. 2020 Apr 30;15(4):e0232494. doi: 10.1371/journal.pone.0232494 (PMC7192485; doi:10.1371/journal.pone.0232494)
Supplement: Attachment — Submitted filename: Response to Reviewers041420.docx [file pone.0232494.s002.docx]

April 13, 2020

To: Demetrios G. Vavvas

PLOS ONE

RE: A Semi-Automated Machine-Learning Based Workflow for Ellipsoid Zone Analysis in Eyes with Macular Edema: SCORE 2 Pilot Study

Ref.: PONE-D-19-34185R1

Dear Demetrios G. Vavvas,

We appreciate the thoughtful reviews to enhance the quality of our manuscript for consideration of acceptance in PLOS One. Please see our responses below which address each of the points outlined by the reviewers.

PONE-D-19-34185R1
A Semi-Automated Machine-Learning Based Workflow for Ellipsoid Zone Analysis in Eyes with Macular Edema: SCORE2 Pilot Study
PLOS ONE

Dear Dr Domalpally,

Thank you for submitting your manuscript to PLOS ONE. After careful consideration, we feel that it has merit but does not fully meet PLOS ONE’s publication criteria as it currently stands. Therefore, we invite you to submit a revised version of the manuscript that addresses the points raised during the review process.

The manuscript has improved and there are some minor points remaining to be addressed. We look forward to the revised version

We would appreciate receiving your revised manuscript by May 28 2020 11:59PM. When you are ready to submit your revision, log on to <https://www.editorialmanager.com/pone/> and select the 'Submissions Needing Revision' folder to locate your manuscript file.

If you would like to make changes to your financial disclosure, please include your updated statement in your cover letter.

To enhance the reproducibility of your results, we recommend that if applicable you deposit your laboratory protocols in protocols.io, where a protocol can be assigned its own identifier (DOI) such that it can be cited independently in the future. For instructions see: <http://journals.plos.org/plosone/s/submission-guidelines#loc-laboratory-protocols>

**Reply:** Our protocol is located with the KNIME workflow uploaded to the KNIME Hub and is completely open access. The link has provided in the data sharing component of the submission: https://hub.knime.com/tetheridge/spaces/Public/latest/Raw_Thickness_Maps

Please include the following items when submitting your revised manuscript:

- A rebuttal letter that responds to each point raised by the academic editor and reviewer(s). This letter should be uploaded as separate file and labeled 'Response to Reviewers'.
- A marked-up copy of your manuscript that highlights changes made to the original version. This file should be uploaded as separate file and labeled 'Revised Manuscript with Track Changes'.
- An unmarked version of your revised paper without tracked changes. This file should be uploaded as separate file and labeled 'Manuscript'.

Please note while forming your response, if your article is accepted, you may have the opportunity to make the peer review history publicly available. The record will include editor decision letters (with reviews) and your responses to reviewer comments. If eligible, we will contact you to opt in or out.

We look forward to receiving your revised manuscript.

Kind regards,

Demetrios G. Vavvas
Academic Editor
PLOS ONE

[Note: HTML markup is below. Please do not edit.]

Reviewers' comments:

Reviewer's Responses to Questions

**Comments to the Author**

1. If the authors have adequately addressed your comments raised in a previous round of review and you feel that this manuscript is now acceptable for publication, you may indicate that here to bypass the “Comments to the Author” section, enter your conflict of interest statement in the “Confidential to Editor” section, and submit your "Accept" recommendation.

Reviewer #1: (No Response)

Reviewer #2: (No Response)

2. Is the manuscript technically sound, and do the data support the conclusions?

The manuscript must describe a technically sound piece of scientific research with data that supports the conclusions. Experiments must have been conducted rigorously, with appropriate controls, replication, and sample sizes. The conclusions must be drawn appropriately based on the data presented.

Reviewer #1: Yes

Reviewer #2: Yes

3. Has the statistical analysis been performed appropriately and rigorously?

Reviewer #1: Yes

Reviewer #2: Yes

4. Have the authors made all data underlying the findings in their manuscript fully available?

The [PLOS Data policy](http://www.plosone.org/static/policies.action#sharing) requires authors to make all data underlying the findings described in their manuscript fully available without restriction, with rare exception (please refer to the Data Availability Statement in the manuscript PDF file). The data should be provided as part of the manuscript or its supporting information, or deposited to a public repository. For example, in addition to summary statistics, the data points behind means, medians and variance measures should be available. If there are restrictions on publicly sharing data—e.g. participant privacy or use of data from a third party—those must be specified.

Reviewer #1: Yes

Reviewer #2: Yes

5. Is the manuscript presented in an intelligible fashion and written in standard English?

PLOS ONE does not copyedit accepted manuscripts, so the language in submitted articles must be clear, correct, and unambiguous. Any typographical or grammatical errors should be corrected at revision, so please note any specific errors here.

Reviewer #1: Yes

Reviewer #2: Yes

6. Review Comments to the Author

Please use the space provided to explain your answers to the questions above. You may also include additional comments for the author, including concerns about dual publication, research ethics, or publication ethics. (Please upload your review as an attachment if it exceeds 20,000 characters)

Reviewer #1: The authors opted to make no changes on comment #3. Regarding their answer:

1. In their article entitled Spectral-domain optical coherence tomography (SD-OCT) patterns and response to intravitreal bevacizumab therapy in macular edema associated with branch retinal vein occlusion, Kang et al found that the integrity of the EZ before treatment could not be evaluated in cross sectional OCT scans in 8 of 67 patients with BRVO-ME.
Herein the authors by converting cross sectional images into en face report >90% of baseline images were ‘ungradable’. This is vastly different from the ungradable percentages in cross sectional OCT. Thus it seems the inability to accurately assess the EZ is not an inherent challenge in RVO (as the authors response to the comment suggests) rather an inherent limitation of en face images, which should be clearly stated in the limitations.

**Reply:** Thank you for the comment. Both central and hemi-retinal vein occlusion have significantly more hemorrhage and fluid than branch retinal vein occlusion, making a comparison between the disease processes problematic. The SCORE2 study data were obtained from eyes with macular edema secondary to central or hemi-retinal vein occlusion. To further emphasize the challenge inherent in evaluating the ellipsoid zone on SD-OCT scans in central and hemi-retinal vein occlusion, regardless of cross sectional or en face evaluation, the authors have include a new figure (Figure 3 lines 217, 286, 288, and 478-481) depicting a representative cross sectional B-scan at baseline with significant hemorrhage and fluid blocking the outer retinal signal intensity to the point of being unable to identify the retinal pigment epithelium. The figure also includes a follow-up month 1 cross sectional image after resolution of a majority of hemorrhage and fluid with intravitreal anti-VEGF therapy and gradable ellipsoid zone.

Change made to manuscript: **Figure 3.** (**A**) Representative center point SD-OCT scan deemed ungradable at baseline for ellipsoid zone (EZ) assessment. EZ was considered ungradable when the retinal pigment epithelium was not visible due to signal blockage by hemorrhage and edema. (**B**) Month 1 follow-up SD-OCT scan after single intravitreal anti-VEGF injection. EZ considered gradable.

2. A prognosticator available at 1 month is not as useful to clinicians and patients as baseline prognosticators for long term functional outcomes in cases those exist. In BRVO the EZ integrity at baseline is a well established prognosticator that the en face approach seems to fail to offer.
All 3 articles that the authors cite in their response refer to cross sectional OCT studies and 2/3 actually did evaluate EZ disruption at baseline with no mention on the potential effect of macular edema. Hence, it seems that the en face transformation of cross sectional OCT images limits the ability to assess the EZ integrity due to macular edema and/or haemorrhage.

**Reply:** Thank you for the comment. The studies by Chan et al, Tang et al, and Fujihara-Mino et al (below) included eyes with central retinal vein occlusion and branch retinal vein occlusion. As stated above, comparing the two distinct disease processes is problematic owing to the significant difference in hemorrhage and fluid in central retinal vein occlusion compared to branch retinal vein occlusion. In addition, these studies used high resolution SD-OCT scans, whereas the SCORE2 clinical trial did not to add generalizability to the study methods and results. This was included in the discussion of our studies limitations (lines 314-320).

The following change has been made to the manuscript ( line 324): *Studies have examined the association of EZ integrity at baseline as a predictor of future visual acuity.^30-32^ However, these studies included eyes with branch retinal vein occlusion, which presents with significantly less hemorrhage and fluid compared to central and hemi-retinal vein occlusion, especially within the CSF.^1^*

3. Further, the authors reply to the comment suggesting that histopathological studies would be needed to determine whether EZ defects on SD-OCT are artifacts resulting from signal blockage or loss of photoreceptors secondary to disease. However the article by Kanakis et al they are citing in the relevant part of their discussion (lines 282-298) describe the development of ‘a method of en face representation of the ellipsoid zone, along with the removal of shadows, to evaluate the ellipsoid layer disruption’ using ‘a MATLAB (Mathworks, Inc, Natick, MA) implementation of the algorithms for shadows removal’ concluding that ‘It could be assumed that the defects at the level of photoreceptors could be the result of the coexisting macular edema. However, the concordance of the ischemic area as shown in FA with the area of ellipsoid disruption at the en face reconstruction of the OCT makes this hypothesis unlikely.’
Good quality images are crucial in Machine Learning. The authors herein employed Machine Learning and transformed the cross sectional images to en face ending up >90% of baseline images were ‘ungradable’in terms of the main parameter the authors seek to study. To make their study stronger, and given their machine learning expertise, I would suggest the authors should embrace a similar approach as the en face OCT article by Kanakis et al they are citing and supplement their methods by developing an algorithm for artifact removal so that the quality control of the images they are feeding the Machine Learning is improved.

**Reply:** Thank you for the comment. Similar to the studies by Chan et al, Tang et al, and Fuijihara-Mino et al, the aforementioned study by Kanakis et al was performed using high resolution SD-OCT scans not obtained for use by clinical trials or clinical practice. The SCORE2 SD-OCT scans were not high-resolution. In addition, the study by Kanakis et al only utilized Spectralis SD-OCT machine. The SCORE2 trial utilized Spectralis and Cirrus SD-OCT machines. Therefore, our methods permitted the use of either imaging device. This limitation was included in the discussion (lines 320-321).

Change made to manuscript: *These studies also utilized high resolution SD-OCT scans, whereas the SCORE2 trial did not to improve the generalizability to the study methods and results. Although post-image processing to remove shadowing cast by hemorrhage and fluid has been performed,^27, 33^ these studies have also utilized high resolution SD-OCT scans from a single SD-OCT device. Our method combined multiple SD-OCT machines.*

Reviewer #2: Almost all my concerns are answered clearly.
Just to be sure, please let me confirm it.
(1) Methods section (page 8, line 175-179). "Total dataset" comes from SCORE2 data. So, it didn't have normal subject data. Is it correct?

**Reply:** Thank you for the clarification. The total dataset comes from SCORE2 data and therefore does not include data from “normal” or “healthy” subjects. This clarification was made in the methods (lines 177-179).

Change made to manuscript: *All scans were obtained from the SCORE2 dataset and were therefore from eyes affected by either CRVO or HRVO.*

7. PLOS authors have the option to publish the peer review history of their article ([what does this mean?](https://journals.plos.org/plosone/s/editorial-and-peer-review-process#loc-peer-review-history)). If published, this will include your full peer review and any attached files.

If you choose “no”, your identity will remain anonymous but your review may still be made public.


**Do you want your identity to be public for this peer review?** For information about this choice, including consent withdrawal, please see our [Privacy Policy](https://www.plos.org/privacy-policy).

Reviewer #1: No

Reviewer #2: No

Sincerely,

Amitha Domalpally, MD, PhD

Fundus Photograph Reading Center

Department of Ophthalmology and Visual Sciences

University of Wisconsin School of Medicine and Public Health
